# Human enhancement, past and present

**DOI:** 10.1007/s40592-025-00250-5

**Published:** 2025-05-27

**Authors:** Andrew Moeller, Jose Maria Andres Porras

**Affiliations:** 1 University of Oxford, Oxford, UK; 2 University of North Carolina at Chapel Hill, Chapel Hill, USA

**Keywords:** Enhancement, Eugenics, History, Life extension, Medieval alchemy

## Abstract

One important role the medical humanities might and should play relates to public education. In this instance, we mean helping persons to think about their own aims or purposes as potential receivers of enhancement interventions, and similarly helping to inform the developers of said interventions. This article argues that, in the light of real and speculative applications of emerging biotechnologies and artificial intelligence aimed at human enhancement—including germline genetic engineering, the linking of the human brain with an artificial general intelligence by way of a brain-computer interface, and various interventions directed toward life extension—historians would do well to consider the following three practices as they participate in the medical humanities and the shared task of public education: (1) Taking under scrutiny a broad swath of topics and timeframes as it relates to past efforts aimed at human enhancement; (2) Focusing on past engagement with enhancement efforts and their perceived relation to the pursuit of living well; and (3) Entering into debates on enhancement as equal participants. In support of these assertions, this article takes efforts directed towards the prolongation of life in medieval Europe as an illustrative example. It also highlights continuities and discontinuities between past and present justifications for human enhancement, and addresses how similarities and differences can shape and challenge contemporary bioethical arguments.

## Introduction

This article is concerned with human enhancement, past and present. In the present, a broad set of developments across the fields of artificial intelligence and biology are unlocking transformational powers over the natural world.^1^ Set within that context, a slew of proposals directed at the enhancement of humans have gained notoriety in recent years. These include selecting for specific traits through germline genome editing (GGE), linking or merging the human brain with artificial intelligence, and various proposals associated with life extension. Imminent and aspirational proposals deserve attention from medical humanities scholars because these proposals foreground questions of embodiment, meaning, and purpose that are of perennial interest to the humanities. Put another way, they prompt general considerations on living well and human limitations, and also reflections on whether particular cognitive and physical enhancements would help persons to live well as individuals and members of communities.

In light of the broader contexts outlined above, historians would do well to consider three practices as they participate in the medical humanities, and specifically engage in interdisciplinary discussions over proposals for human enhancement, as well as the related and overarching discourse on meaning and purpose. First, given the variety of human enhancements being pursued in the present day, we recommend that historians intentionally draw upon an expansive set of topics and periods. Next, we recommend that historians attend to past reflections on human enhancement and its relation to the existential pursuit of living well. Finally, we recommend that historians enter into contemporary debates and discussions over the right uses of emerging technologies as equal participants with their non-historian peers.

In support of the above assertions, this article takes efforts directed towards life prolongation in medieval Europe as an illustrative example. It further highlights continuities and discontinuities between past and present efforts aimed at enhancement, and addresses how these similarities and differences can challenge and shape contemporary debates. We have in mind that such debates would ultimately have an eye towards public education. Historians would thus deliberate with, and learn from, their peers from a variety of disciplines under the shared umbrella of the medical humanities. Upon having their ideas challenged and strengthened, all in turn might better serve individuals and communities in their respective processes of making wise decisions regarding the development and adoption of enhancement technologies.

## Drawing upon expansive sets of topics and timelines

One familiar use of history as it pertains to deliberations over human enhancement is drawing from late-nineteenth- and early-twentieth-century eugenic thought and practices to inform moral assessments of modern-day technologies like GGE that could allow for control over inborn traits (Sandel 2007; Sparrow 2011; Friedmann 2019). One can define eugenics in a historic sense as a broad grouping of beliefs and both ‘positive’ efforts (i.e., encouraging the so-called ‘fit’ to procreate) and ‘negative’ efforts (i.e., restricting procreation amongst the so-called ‘unfit’) aimed at improving the inborn qualities of particular groups of people or, more generally, of societies.^2^ Eugenic thought and practices in the United States and Europe during the first half of the twentieth century are regularly used as reference points in contemporary debates, but historians have helpfully revealed the pervasiveness of eugenics outside of those regions (Chung 2014; Turda and Gillette 2014). Scholars like Alison Bashford have also shown that eugenic practices and ideals persisted long past the Second World War (2012, 542–551).

Yet, no matter how far the eugenics timeline is extended toward the present, or how widespread eugenics is discovered to have been, it still holds that many contemporary proposals for enhancement procedures cannot be readily compared to the biological means of enhancement promoted by nineteenth and twentieth-century eugenists. For example, epigenetic reprogramming in adults for the purpose of reversing or inhibiting aging has little to do with the selection for inborn traits.^3^ The same holds true for technologies aimed at linking the human brain with AI (Lyreskog et al. 2023, 13–14). The more limited applicability of the history of eugenics to deliberations over some forms of human enhancement serves as an encouragement to conduct and draw upon historical research relating to past topics and themes that might function as better parallels and bring useful insights.

We in no way mean to devalue the importance of the history of eugenics as it pertains to contemporary debates over certain enhancement proposals or overarching conversations relating to meaning and purpose. Instead, we commend that a much wider history of enhancement (into which many eugenic schemes and practices might be placed) be taken under scrutiny.^4^ That wider history encompasses thousands of years of diverse efforts by human beings to overcome or transcend the bounded human form. Included were projects specifically aimed at life extension.

We contrast enhancement with therapeutic medicine aimed at treating conditions like diseases or injuries, and define the former as the improvement of'human capacities, performances, dispositions, and well-being beyond the traditional scope of therapeutic medicine… Enhancements aim to augment a desirable capacity that is already within the normal range for our species' (Giubilini and Sanyal 2015, 233). This definition naturally raises questions relating to embodiment, meaning, and purpose that are of historical and ethical importance, such as why any particular capacity or trait might be deemed desirable.

No single historian could take up the project of exploring the extensive history of human enhancement across all regions and time periods.^5^ Numerous historians from many different specialties must instead share the task of examining particular and applicable past efforts aimed at enhancement. We have chosen to provide relevant examples from Western Europe during the late Middle Ages because one author of this paper specializes in the history of medicine, theology, and natural philosophy during that period.

### The medieval pursuit of the prolongation of life

Perhaps nowhere in the past have science and technology had such a pervasive and transformative effect on society as compared to today. But science and technology have always been part of human culture, and efforts aimed at leveraging them to overcome the human condition were not strange in medieval Europe. Medieval scholars often pondered and experienced the precariousness of human life. In the book of Job (14:1–2), they read how ‘man who is born of woman is of few days and full of trouble. He comes forth like a flower and fades away; he flees like a shadow and does not continue.’ The fleetingness of life, and other associated evils of sickness, infirmity, and separation by death were thought to be principally the result of the Fall, that is of Adam and Eve’s rebellion against God. Such theological rationalization, however, did not stop medieval thinkers from trying to find ways to ameliorate, if not to remedy, the shared human conditions of aging and mortality (De Rijk 1967).

Accordingly, desires for a long and blissful life appear in various medieval texts and genres. The widely disseminated ‘Letter of Prester John’ played with ideas of an imaginary and utopian realm populated with fountains that restored life. There all physical and moral decay were absent. This letter fascinated those in the highest circles of the papal curia in the twelfth century (Paravicini Bagliani 2000, 199). Legends of Alexander the Great also spoke of three miraculous fountains: one capable of turning death into life; one of conferring immortality; and the last of restoring youth (Ibid., 203).

Moving into the thirteenth century, we witness the emergence of scientific treatises that circulated with titles like *On Delaying Old Age* (*De retardanda senectute*).^6^ As numerous scholars have shown, within these later medieval works, the key to achieving the prolongation of life (*prolongatio vitae*) was not found in inaccessible prelapsarian worlds, exotic geographies, or magical fountains but, rather, in the manipulation and perfection of nature by science and technology (Ziegler 2001; Crisciani 2009; Allen 2023). It is in this sense that some modern-day attempts at achieving life extension can be considered a continuation of the project of medieval alchemists. For the aim of medieval alchemy was primarily the perfection of nature by technique and, indeed, one common pursuit of many alchemists was the prolongation of human life.

In medieval medicine, health was understood as a process of restoration of the equilibrium (*aequalitas*) of the humors of the body. Humors, in turn, determined one’s bodily complexion.^7^ Original sin had supposedly disrupted this equilibrium, but so also did the subsequent ‘sins’ of men who, amongst other faults, had not kept to the proper habits of living and eating (*regimines*). Thus, the Franciscan philosopher, scientist, and theologian Roger Bacon (1220–1292) ascribed the shortening of life after the Fall to human stupidity (*stultitia*) which led to the degeneration of the human body and, through reproduction, more and more degeneration amongst subsequent generations. Bodily degeneration also contributed to drying up the radical moisture (*humidum radicale*) which, ultimately, led to death (McVaugh 1974; Crisciani 2010). Hence, as Meagan S. Allen has put it, for Bacon, ‘almost all men died by potentially remediable accidents before the time appointed to them by God’ (Allen 2023, 185).

The shortening of life was sometimes regarded as a divine punishment fitting to the fallen human condition, assuring that humans would not fester in their sin for too long. However, even then, the longevity of the first generations between Adam and the Flood, as well as the promise of the bodily resurrection at the end of time, demonstrated that the human body could be improved—and life span extended or at least restored. If the human body was close to being incorruptible before the Fall, and would be fully incorruptible at the end of time upon the return of Christ to this earth, then there was no reason why it could not get closer to incorruptibility in this life (Ibid., 201). The same theological rationale that explained the precariousness of human life undergirded the optimism about man’s ability to prolong his life–by means of medical and alchemical remedies–amongst theologians. By contrast, physicians tended to distrust any belief in naturally caused immortality (Ziegler 2001, 234).^8^

### Contemporary religious engagement with life extension

Neither the Christian Scriptures, nor any major branch of Christianity, insists on a particular view relating to life extension. A common conclusion drawn by contemporary Christian thinkers on life extension is that of ambivalence (Daly 2021), but at least one prominent Christian bioethicist, Greg Meilander, has staked out a more critical stance on the practice. In part, Meilander suggests that (indefinite) life extension could never provide Christians with the deepest desire of their hearts, that being the direct enjoyment of God for all of eternity (Meilander 2013).

Here the history of moral reasoning in favor of life prolongation in the Middle Ages might challenge condemnatory positions in at least two ways. First, every major branch of Christianity assigns considerable significance and authority to tradition and the history of Christian thought as it pertains to adjudicating ethical problems in the present. The views of Bacon and others in the medieval period suggest that it could be Christian critics of life extension in the present who stand out of step with the history of Christian moral reasoning on the topic. Second, and as will be discussed more fully later in this article, medieval proponents of life prolongation did not view the practice as a means to frustrate or delay a final and permanent union with God, but to anticipate and prepare for it.

As it pertains to broader bioethical deliberations that are not limited to any one religion, the persistence of specific arguments in favor of life extension should not mechanically lead to the conclusion that such arguments bear more weight than others. However, the temporal persistence of certain arguments, especially across religious and metaphysical divides, can and should engender serious consideration of them. For example, Christian proponents of humans living longer lives in the past (such as Bacon) drew attention to how more years on earth might contribute to a process of meaningful personal development–which is a line of thought that is also potentially relevant to, as well as promoted by, non-believers in the present day (Linden 2022). This might encourage those in the present who are eager to paint radical life extension as inimical to a life of purpose and direction to consider if they may have been too quick in reaching such conclusions (Kass 2002), or at least if they have fully considered relevant counter-arguments.

Historians might also look to different regions and time periods to examine past efforts relating to life extension—including Russian cosmism in the early twentieth century (Young 2012). Scholars seeking to contribute to other discussions, like linking the human brain with AI, could potentially examine past efforts to transcend the limitations of the human mind, including practices relating to astrology and other forms of divination (Wisniewski 2020). Those practices might serve to inform the present by drawing attention to how various forms of purported enhancement in the past were scientifically questionable, which can prompt consideration as to whether the same could be true for forms of enhancement proposed in our own time. Another benefit of such studies would be assessing the motivations that drove similar endeavors in the past and, in particular, how they related to various conceptions of living well. To this, we now turn.

## Human enhancement and living well

It may be that the project of (radical) life extension is never realized. Or that, along with other proposals for human enhancement, extending the human lifespan by many decades or centuries will correspondingly not be achieved for decades or centuries. Such proposals are still worth reflecting on and assessing because scientists, engineers, and others must today make decisions regarding how and if they will contribute to research and development, and also because, however small the possibility, these technologies might one day be widely available to the public. As evidenced by the transformational impacts of the advent of the internet and smartphones (Carr 2011; Sohn et al. 2019), we would benefit from reflecting on the desirability of emerging technologies prior to their widespread adoption.

There are numerous, overlapping factors that individuals, families, and communities can and should take into consideration when evaluating enhancement proposals. Matters of identity, autonomy, and privacy are all often relevant (Lyreskog et al. 2023, 13). So too may be fears over short and long-term physical side effects. Then there are considerations that fall more squarely into the realm of what might be termed existential values. Within that latter sphere, there are ongoing and long-standing conversations over technology, the body, and human meaning and purpose. For example, Nick Bostrom wrestles with these matters while imagining a future of significantly enhanced (and potentially disembodied) human beings:

Suppose that we develop superintelligence safely, govern it well, and make good use of the cornucopian wealth and near magical technological powers that this technology can unlock… Here we confront a challenge that is not technological but philosophical and spiritual. In such a solved world, what is the point of human existence? What gives meaning to life? (2024).^9^

Another way to frame the immense questions of meaning and purpose is to ask: would the adoption of an enhancement help us to live well as individuals and members of communities?

We use the term'live well' to refer to what a person might deem a superior way of being—or a way of experiencing, relating to, and interacting with oneself, other persons, the natural world, and self-recognized supernatural realities. Questions pertaining to enhancement and living well need to be answered partly in the light of conceptions of the shared physical nature of human beings (or in the light of the fact that we are corporeal or embodied creatures), our purpose or purposes, our place in the natural order, and attending conceptions of flourishing. Naturally, when reflecting on the relationship between enhancement technologies and living well, one is also prompted to reflect on which good the adoption of a particular technology would serve to promote, and how the pursuit of that good would reflect or rearrange one’s own ordering of goods.

We are not advocating for a relativist account of living well, nor an account detached from empirical research. What we are suggesting is that deciding for oneself what constitutes the superior way of being in the world is one of the great tasks of life, and one that each person must ultimately decide on for themselves. The discipline of history can contribute to efforts aimed at public education by drawing upon hundreds and even thousands of years of reflections across philosophical and ideological traditions on what it means to live well and how technologies might aid or hinder such pursuits by overcoming or extending the bounded human form. This includes identifying both fruitful and pernicious lines of thought.

### Medieval goals for life extension

A cursory glance at medieval alchemy and the work of figures like Roger Bacon might suggest only esoteric interest in the magical properties of natural elements and their ability to transform the human body. A deeper look, however, reveals profound interests in conceptions and pursuits of living well. We may then ask: for those medieval thinkers who believed it possible to extend the human lifespan, what were their ultimate goals? That is, how could the prolongation of life aid the pursuit of living well (or what would have been referred to as the ‘good life’)?

Notably, what might be termed practical concerns relating to living well were not absent in medieval thought. In one popular work called the *De retardatione*, the prolongation of life found its *raison d’être* in between the private and the public sphere. In it, the well-being of the sovereign’s body was strongly linked with the prosperity of the community over which he presided. Consequently, such academic works on the prolongation of life found an audience within the imperial and papal courts of the thirteenth century. Also of note, by that time new political manuals had placed a stronger emphasis on the aesthetics of rule which included care for the sovereign’s external appearance (Paravicini Bagliani 2000, 197). Hence, medieval history shows that efforts at achieving prolongation of life, or retardation of old age, must also be considered in the context of political ideas and ideologies of rule.

However, for many medieval thinkers, the pursuit of a longer life was often directed toward explicitly spiritual ends. In the Middle Ages, sciences aimed at the prolongation of life, like medicine, astronomy, and alchemy, were as much an art (*ars*), conceived of here as trying to live in accordance with Christian moral principles, as a science (*episteme*) or mere technique (*techne*). Life prolongation was co-terminus with the perfection of one’s body and physiological complexion, that is making one’s body as close as it could be to the body one could expect to receive from God after the resurrection. According to Bacon, this body, in turn, could contribute to perfecting the soul, its wisdom and virtues, and its ability to learn the sciences (*ex nobilitate complexionis excitaretur et vigoraretur anima rationalis in tantum ut possit de facili scire omnes scientias*). Similarly, the prolongation of life, and the resulting improvement of one’s intellectual faculties, supposedly had the potential to make the individual better able to rule himself and others (*ut homo deveniat ad magnam prudentiam et sapientiam perfectam ut sciat se et alios regere*) (Paravicini Bagliani 2003, 50). So then, physical infirmities and death were not the only, or even main, negative effect of Adam’s Fall that medieval science sought to remedy. The hope was to find solutions to address human ignorance and concupiscence, imperfections of the intellect and the will, which were thought to pose far greater dangers than physical ailments to the fate of one’s soul.^10^

The perfecting of one’s body could also help increase man’s knowledge of natural things and this, in turn, could lead him to greater union with God. In the mind of the Dominican Giles of Lessines (c. 1260), the longevity of the first generations of men after the Fall had been meant by God to ‘allow the accumulation of a critical mass of diverse speculative and experimental knowledge of nature (*scientia rerum naturalium*), especially astrology, necessary to ascend to the knowledge of God’ (Ziegler 2017, 334).

The prolongation of life, and the concomitant perfection of one’s body and mind, was also said to assist a process of corporeal deification. This idea was part and parcel of the spirituality of Franciscan friars, like Bacon, who went as far as to endorse the prolongation of life as a quest for physical oneness with the divine (French and Cunningham 1996, 208, 223−36). Bacon himself surmised that the human soul, thought incorruptible, is unhappy being contained in a corruptible body (Allen 2023, 189). Thus, life prolongation efforts could contribute to alleviating the tension between body and soul. That process would result in a greater identification with the resurrected Christ and through Him with the divinization of the Christian individual. However, all of this, including the attainment of immortality, could not be perfectly achieved before the resurrection. Until that time arrived, it was through their participation in the Eucharist that Christians were made like Christ, and incorporated into His mystical body (Allen 2023, 194).

Most medieval authors believed that while one’s demise could be delayed, and the infirmities of old age rendered more bearable, death nevertheless remained natural and inevitable. Indeed, it was commonplace to emphasize that the goal of medicine and alchemy was not immortality, or lengthening life spans at any cost, but to reach the maximum age allotted by God and nature. Medieval treatises on the prolongation of life reveal a desire to develop and perfect the human person, understood as a body-soul unity oriented towards virtuous behavior and destined to participate in the divine life through grace. The perfection of the human body was only the first step towards the full realization of the human person because, as Thomas Aquinas put it, ‘grace does not remove but perfects nature’ (*Summa theologiae* Ia q. 1, a. 8 ad 2). It is only with this in mind that one can understand why medieval thinkers like Bacon held that science had to be at the service of divine truth (Bacon 1928, 65−7, 72−4) or, as it was often put, that philosophy was the handmaid of theology (Grant 1996, 1–17).

### The applicability of historical accounts of living well

Historical explorations of specific instances of pursuing human enhancement for the purpose of aiding conceptions of living well can prove to be relevant to correlated technological projects today, such as is the case with life extension in the past and present. For example, we could note that medieval thinkers believed life extension was desirable not simply for the attainment of some general idea of growing in knowledge and moral virtues, but ultimately for better knowing and serving God. Whether or not that last point is should be deemed true or desirable in the present is open for debate, but it is a compelling line of argument that billions of religious people across the world might want to consider for themselves as it relates to enhancement, meaning, purpose, and embodiment (i.e., limitations relating to longevity, health, and the human body).

Here we can also highlight discontinuity between the past and present. Comparing the views of Nick Bostrom to medieval proponents of life prolongation, we see several major points of departure regarding embodiment and the importance assigned to technology. Bacon, in line with the majority opinion amongst medieval alchemists, did not want to escape from the given limitations of embodiment, nor did he think he could escape mortality. As mentioned, Bacon presumed one might be able to live longer through alchemy (in accordance with the limits assigned by God), but the science of alchemy could not provide immortality. Furthermore, he viewed the physical body as essential to human identity, key to human flourishing, and as a good gift from God. Bacon thus understood true immortality as only possible through the power of God at the end of history, at which time human beings would receive an incorruptible body like that of the resurrected Christ as well as, critically, a purified soul that would be united in total harmony with such a body. They would then flourish for all of eternity as embodied beings.

In contrast, Bostrom holds that it might be possible for humans to achieve a near or approximate immortality based on their own efforts–and he presents a view of the givenness of the human body as a kind of hindrance to the fulfillment of human aspirations and deep human longings (Bostrom 2024). In a sense, humans might bring heaven to earth (though Bostrom suggests we may also inaugurate a kind of hell) through the transcendence of bodily limitations.

The views of Bacon, then, also stand in contrast to Bostrom’s notion of the role technology might play in bringing about a “solved world.” For Bacon, technology had instrumental value in so far as it helped men and women to love both God and neighbor, and relatedly to live better lives by improving the physical condition of the body and the use of intellectual and moral faculties of the soul. But the fundamental problems thought to be facing humanity, including the prevalence of evil and other consequences of the Fall, were moral and spiritual and not solvable through technological means but needing, instead, the right use of free will and the help of God’s grace. The notion of Bostrom’s “solved world,” in contrast, assigns apocalyptic-like dimension to its assessment of the potential efficacy of enhancement technologies. In other words, humans might very well be able to solve nearly every problem we face through the power and correct application of technology. This optimism can encourage a framing of the problems faced by human beings principally in terms of technological research and development, potentially obscuring the non-technological dimensions of the challenges we face as a society.

Bacon might also challenge views similar to those of Bostrom by raising the matter of if and how human embodiment, or the givenness, and especially limitations, of the human form, plays a central and necessary role in human flourishing, and also what might be said to be the moral significance of the given human form. Following this line of thought, the past may shed light on the risks posed by contemporary efforts aimed at human enhancement. In this instance, not necessarily in regards to negative physical and mental side effects, but in a potential subversion of our shared identity as bounded creatures and related common flourishing.^11^

Notably, we can also find shared aims between different technologies in the past and present. One might usefully draw on a figure like Bacon, or a twentieth-century eugenic figure like Julian Huxley, in assessing and determining the wise applications of linking the brain with AI. For his part, Huxley promoted a notion of living well in a 1941 collection of essays that stressed the attainment of profound experiences in fields such as the arts, sciences, literature, and even human relationships. For example, there was thought to be considerable moral value in the experiential aspect of solving the scientific mysteries of the universe, but also in areas like giving and receiving romantic love. Through the “science” of eugenics, Huxley (who took up a similar, albeit distinct line of thought from Bostrom) presumed that the genetic capacities of human beings could perpetually advance, thus also unlocking newer and more profound experiences for human beings. In the case of brain-computer interfaces, one could ask if it would be wise to pursue that conception of living well by a different route. Namely, transcending our embodiment—and so visiting beautiful locations (both on and off planet) from the comfort of one’s own sofa.

Looking to Bacon, it can be debated as to the extent the use of a BCI would generally align with his accounts of living well, for example by providing near instant access to seemingly infinite knowledge, or even by allowing immediate access to a digital ‘moral guru’ for the purpose of making moral decisions (Lyreskog et al. 2023).^12^ As to that latter point, one would need to consider that Bacon possessed a different conception of what constitutes a moral way of living as compared to an account that would focus only on performing correct moral actions and is less concerned with the development of virtuous moral inclinations in the will. Considering both Bacon and Huxley, their accounts of living well can also serve more generally discussions on enhancement, embodiment, meaning, and purpose.^13^ One can ask, for example, whether profound experiences are central to conceptions of living well.

Past accounts of living well can also engender critical reflection on the prioritization of resources in the present. For example, some proponents of life extension speak of aging and death as one of the greatest evils facing humanity (de Gray 2007; Linden 2022, 4–5). Hence, radical life extension might be conceived of as the greatest good that can be pursued. Medieval proponents of life prolongation can challenge such views with a different set of priorities. Meaning that for medieval proponents of life extension it would have been morally dangerous to overvalue life prolongation at the expense of other perceived moral duties. The late medieval Church placed charity highest up in the order of goods and it affirmed that no virtue was possible without charity (Aquinas, *Summa theologiae*, IIa-IIae, q. 23, a. 7, co.). This love of neighbor materialised in the establishment of hospitals, leper houses, orphanages and other charitable institutions that provided food, clothing, shelter, and medical care to the poor, the sick, and those on the fringes of society (Brodman 2009). Along these lines, Bacon and his Franciscan brethren also placed a great moral weight on simple acts of charity. Hence, in answer to the question of ‘what do we want to live for?’ many medieval men and women would have answered in part ‘to care for the orphan and widow in their distress.’ Such a view can challenge the time and money that are funnelled into life extension research.

Lastly, medieval reflections on living well, and the context in which those reflections occurred, can foster several questions as to how enhancement technologies might disrupt or impact contemporary societies. As evidenced above, a common framework existed amongst medieval thinkers as to what constituted the good life or living well. Looking to applications in the present day, what pitfalls might come about from life extension in a context without such a shared framework? Can societies flourish in which people live for as long as they like, but share vastly different, antithetical, selfish, or even immoral aims and goals? Might the current political, cultural, economic, and ideological divides we find across contemporary cultures further harden, and ultimately prove intractable and highly corrosive, without new generations of leaders and scholars and activists to challenge value divides?

Clearly, we can benefit from thousands of years of reflections on living well and enhancement. But we can also gain from the reflections of our fellow community members within educational and other contexts—who themselves have likely wrestled with their own relevant conceptions of existential values. As members of the same communities, and in a context where all community members would likely be impacted, we owe it to our fellow community members to listen and learn from one another.

## The historian as equal participant in debates

Historians participating in the medical humanities may be inclined to limit themselves in debates relating to living well and enhancement procedures to supplying historical narratives or historical analysis. One reason for this could be that many historians seek to avoid moral judgments and valuations in their work, as it is sometimes viewed as antithetical to objective or ‘scientific’ investigations. Another reason could be a belief that they are ‘out of their depth’ as it relates to making prescriptive arguments for or against enhancement proposals–as most historians, by definition, are not trained ethicists. As demonstrated above, there is value in supplying historical narratives and historical analysis. Including as it relates to public education and identifying near-inexhaustible prior strands of thought on living well and the pursuit of human enhancement for consideration.^14^

However, if they should choose, we believe the historian has more to contribute to the goal of public education under the umbrella of the medical humanities—including making their own historically informed, normative arguments in relation to enhancement technologies and living well. When speaking of the epistemic benefits historians can supply to contemporary debates, these may include: (1) fostering an appreciation, by way of analogy and comparison to past examples, of how a present practice or technological venture is based upon good or compelling reasons and hence worth keeping/pursuing; and (2) expanding our sense of how we might live as human beings. For example, history can make one more aware of the diversity of ethical regimes operating in the past, ‘the different goods that were taken to be important at various times, and about how these various goods were ranked or prioritized’ (Grimm 2017, 419). This, in turn, offers an opportunity to inhabit a different moral imagination and, from that vantage point, to consider whether our own order of goods might be misguided–or, conversely to appreciate the merit of our own arrangement of goods insofar as we come to think that a prior society was lacking in various ways.

How then might the utility of history be conceived? Importantly, we are not offering a comprehensive account of the utility of history. Instead, we offer up one kind of approach for consideration.^15^ Wherein the utility of historical inquiry can be compared to asking one’s grandparents for marriage advice. Perhaps they have not had the same exact argument as the one between you and your spouse.^16^ Yet they have likely had similar debates and discussions, and can point you towards overlooked perspectives. They also would be free to explicitly express their own opinions and judgments on the matter you are sharing with them. Meaningful, contemporary contributions to debates over emerging biotechnologies can accordingly be found by connecting the past to the present.

When speaking of the utility of history in this paper, we are therefore principally referring to the utility of the work of careful historical analysis and interpretation, and the subsequent interpretation and application of its relevance to the present day. Within that framework, we want to highlight objectivity (or at least the pursuit of objectivity) in the discipline of history as it relates to obtaining knowledge about the past, but also acknowledge the subjective nature of applying that knowledge to the present. Of course, a grandparent, like a historian, might prove to be unhelpful or misapply the past to the present—and so we are not advocating for an unquestioning approach. But rather, to continue the analogy, we want to endorse an openness to learning from (what might prove to be) the wisdom gained from lived experience. Here one should be cautious of a stance in the present that assumes that we have attained a superior knowledge of right and wrong than that of men and women in the past.

One could imagine the historian saying: ‘Here is something I have discovered or learned from my bounded study of history, and here is how and why I think it is relevant to this particular aspect of the ongoing debate, and here is in particular what I hope to accomplish by participating in the debate.’^17^ Historians could of course still choose to limit themselves to trying to inform others of relevant historical strands of thought. Or what they hope to accomplish could be more prescriptive—in that they want others to adopt their position on the desirability of a particular enhancement. Therein we find the need for interdisciplinary debate. No one discipline has a monopoly on considerations pertaining to living well. And of course, scientific and medical expertise, and not only reflections pertaining to the humanities, need to be taken under advisement.

Other contributions of history that fall within the grandparent analogy include: (1) identifying trajectories in the past that may be repeated today (e.g., a likelihood to overestimate the efficacy of a particular intervention); (2) warnings over the construction of societal narratives of belonging and exclusion (i.e., who is welcomed and valued by society); (3) affective storytelling and forewarnings (e.g., a reminder that we are dealing with real people who can really be hurt, and not abstract populations); (4) adapting from the work of Paul (2014, 267–269), the history of the pursuit of some forms of enhancement could also warn against hubris and an overconfident sense of control.

What is essential is not that one tries to shed any bias in the present—but rather that one is first willing to have their interpretations of the past, and their present convictions, challenged and shaped by the actual historical data. This often necessitates acknowledging how one has been shaped and influenced in terms of their own beliefs and presuppositions in the present, and doing one’s best to put oneself into the mind of the historical actors they are studying. That is, how did they see and understand the world, based on their own beliefs and presuppositions (Wright 2019, 101–105). Upon completing excellent historical work, the historian would then be ready to bring that to bear on larger conversations on specific enhancement applications and broader discussions on purpose, meaning, and embodiment. Again, historians need not try to adopt a position of being unbiased in terms of what they believe is the value of their historical analysis or how they hope to influence current debates. But then they must also accept that they are but one voice at the table of those debates.

What the historian and those involved in present-day deliberations must become more comfortable with is a blurring of the boundaries between the role of academic historian and participant in contemporary debates. Meaning historians would do well to familiarize themselves with current debates, and offer up historically informed perspectives as equal participants in those debates. We would particularly commend historians to reflect on how their limited or narrow accounts of various aspects of the history of enhancement might benefit the present.^18^ All this entails, however, a truly interdisciplinary approach to debate and deliberation—where all those working towards a shared effort of public education under medical humanities seek to learn from their peers in a variety of disciplines.

### Medieval science as a model of interdisciplinary engagement

From the medieval period, many of those who engaged in debates over enhancement could serve as models as to how one might approach interdisciplinary engagement within the medical humanities today. As stated above, one of the surprising characteristics of medieval discussions on life prolongation is that they were often authored by theologians. In medieval times, natural philosophy, the precursor of modern science, was part of the curriculum of a bachelor’s degree which anyone aiming to be enrolled in ‘the higher schools’ of law, medicine, and theology had to complete (Grant 1996, 37–49). As a result, theologians were well placed to discuss questions of natural science. Moreover, many theologians in the thirteenth and fourteenth century had also undertaken studies in medical faculties, or at least display in their work a wide knowledge of current medical theory: the Dominican Thomas Aquinas (Jordan 1988) and Roger Bacon (Getz 1977) are just two examples.^19^

Take the example of another Dominican, Engelbert of Admont. He studied the arts (logic and natural philosophy) at the cathedral school in Prague, then continued his studies in Padua with teachers such as William of Brescia (who had in turn studied with the famous physician Taddeo Alderotti in Bologna) and then spent four more years at the Dominican *studium* in Padua where he studied theology. His corpus reflects this interdisciplinary training, and his scholarly output includes works on theology, political thought, music, moral philosophy, and natural philosophy, including a treatise on the causes of longevity before the Flood (Ziegler 2017, 314−15). At least in four occasions in this treatise, he refers to the authority of contemporary physicians or medical works that had been recently translated from the Arabic tradition (Ibid., 321, fn. 18). The historian or others contributing to contemporary debates and discussions need not undergo similar, formal training across disciplines. But they would benefit from familiarizing themselves with debates in other fields and relevant ethical literature, and also of course by collaborating with colleagues across disciplines.

Also of note, medieval theologians were predisposed to discuss questions relating to enhancement and living well because they were in a favorable position to imagine a natural order beyond the one here in the present. Often, this meant looking backwards (to prelapsarian Adam, or the patriarchs and prophets and prediluvian fathers) or forwards towards glorious, resurrected bodies (Ziegler 2001, 203). In many ways, therefore, Christian Scripture and theological dogmas opened rather than closed the door to speculation on improving the human condition. Indeed, as David Noble has argued, the origins of scientific endeavors in the early modern period can be traced back to medieval efforts at restoring the effects of the Fall (1997).^20^ The preeminent role of theologians in discussions such as the prolongation of life meant that these scientific questions were never treated in isolation from philosophical, moral, and theological truths. As such, they were inserted into a narrative of creation, fall, and redemption.

We recognize historians and others participating in the medical humanities ascribe to a spectrum of metaphysical beliefs. However, while our technology may stand to better achieve the dreams of long life than that available to medieval theologians, we are still faced with the same existential question as they: what do we want life for? Answers to that question today may not so neatly fall into the categories of ‘religious’ and ‘nonreligious’ or ‘areligious.’ When taking up the question of how to live well, is a religious claim to be understood as one, or some combination, of the following: (1) that which is based on a sacred text? (2) that which centers around the authority of a Creator? (3) that which appeals to an invisible, spiritual reality outside of the physical world? Could we add another option: (4) that which draws upon a defined set of existential values and/or a robust philosophical anthropology? Our point here is to encourage dialogue across metaphysical and value divides, and also encourage reflection and engagement within the medical humanities (and efforts aimed at public education) on existential values, by flipping the initial question on its head to suggest what most expansive accounts of living well might have in common. Namely, reflections on the significance of our shared embodiment, what the purpose(s) of a human life might be, what the place of humankind is within the natural order, and what the related, fullest conception might be of human flourishing.

## Conclusion

As mentioned, one goal the medical humanities ought to have in mind is that of public education for both developers of enhancement technologies and potential recipients of enhancement technologies and procedures. This education could take many forms. Including the writing of opinion pieces for newspapers or online outlets; speaking at places of religious worship; attending and speaking at professional conferences relating to the sciences and engineering, and creating materials for engaging children and teenagers in their schools.

This article has argued that, based on the wide array of enhancement proposals being pursued in the present day, as well as their relation to perennial questions of embodiment, meaning and purpose, historians can be better prepared to contribute to public education by drawing upon a wide set of topics and timelines as it pertains to human enhancement, paying close attention to past engagement with living well and the pursuit of enhancement, and entering into debates with academics from various disciplines as equal participants.

## Data Availability

No datasets were generated or analysed during the current study.

## Footnotes

1 This language is paraphrased from the website of ‘The Boundaries of Humanity’ project based at Stanford University (Boundaries.com).

2 Such efforts, which included measures like sterilization, the promotion of birth control, and marriage loans, were often intended to be intergenerational.

3 David Sinclair and collaborators suggest that ‘epigenetic reprogramming’ offers a particularly promising path to combating aging (2023).

4 This practice does not need to be limited to professional historians.

5 The general time frame under consideration ought to be extended as far back as historians can find evidence for relevant practices.

6 These works were often mediated by Arabic commentaries, such as Averroes’ commentary on Aristotle’s *On Length and Shortness of Life* (*De longitudine et brevitate vitae*). For a rich bibliography and a list of these works and their attribution, see Crisciani (2013, 90, fn. 14).

7 From the late thirteenth century, medieval thinkers also distinguished between radical complexion (*complexio radicalis*), the individual complexion of every organ, and the complexion of the whole body (Ziegler 2023).

8 See, for instance, the following statement by the Muslim physician Avicenna—whose work was extremely influential amongst Latin Western doctors—in his *Liber canonis* (1507, f. 53ra): ‘the art of preserving health is not an art that makes us safe from death, nor does it lead each body to the ultimate life span that is absolutely possible for a human [being], but it offers security in two things: it prevents putrefaction and it protects the [radical] moisture, lest it quickly deteriorates.’

9 Quotation taken from promotional material on the back cover of the book. For further discussion, see also President’s Council (2003); Sandel (2007); and Agar (2014).

10 As Richard W. Southern pointed out, growing trust in the ability of human reason to perfect the fallen nature of man was a defining characteristic of medieval humanism in the late twelfth and thirteenth centuries (1970).

11 The need for sleep might be considered as a specific example. Would “freeing” the human body from a need for sleep be considered an enhancement? Another way to ask such a question is the following: what role does the human need for sleep play in the holistic flourishing of human beings?

12 Medieval reflection on living well, life prolongation, and moral formation are also relevant to proposals for the moral enhancement of persons by way of pharmaceuticals.

13 Likewise, a historical analysis of medieval debates surrounding the use of physiognomic science in court trials, for instance, could enrich modern-day discussions regarding the use and dangers of AI-powered facial recognition models to predict sexual orientation or political views. For these emerging uses, see Wang and Kosinki (2018) and Kosinski (2021), respectively.

14 Moreover, by analysing the social, cultural, and political contexts of moral positions in the past, historians can provide those engaged in contemporary debates with resources to better understand moral frameworks that might undergird opposition to enhancement proposals. This can be helpful within pluralistic societies, in which many moral frameworks can and are drawn upon; leaving room for potential confusion and misunderstanding.

15 For further discussion on the utility of history to contemporary bioethical debates, see Wilson (2011); Pernick (2008); and Lerner and Caplan (2016).

16 Historian Diane Paul suggests that drawing one-to-one correlations between present-day applications of biotechnologies and historic eugenic practices can be problematic because'the present always differs from the past in multiple ways.' Another reason is that'historical evidence does not speak for itself—it has to be interpreted' (2014, 266–267).

17 The analogy of speaking with a grandparent, while created by ourselves, was influenced by the work of Paul Ricoeur (1990, 52–90).

18 This would help historians to avoid a kind of ‘death by a thousand qualifications’ that might occur when, for example, trying to provide a sweeping history of eugenics and account for its relevance to today.

19 For a detailed analysis of the relationship between medicine and theology in the medieval university, see Jacquart (2010). See also Ziegler (1999). It is important to note that medical doctors themselves often undertook studies in theology (Courtenay 2001) even if, in their scientific works, they usually avoided engaging in theological debates.

20 See also the remarks in Crisciani (2013, 106) and Cady (2011).

## References

[CR1] Agar, Nicholas. 2014. *Truly Human Enhancement: A Philosophical Defense of Limits*. Cambridge, Mass.: MIT Press.

[CR2] Allen, Meagan S. 2023. *Roger Bacon and the Incorruptible Human, 1220–1292: Alchemy, Pharmacology and the Desire to Prolong Life*. Cham: Palgrave Macmillan.

[CR3] Avicenna. 1507. *Liber canonis Avicenne revisus et ab omni errore mendaque purgatus, summaque cum diligentia impressus*. Venice: Girolamo de’ Paganini.

[CR4] Bacon, Roger. 1928. *The Opus Majus of Roger Bacon*, vol. 1, trans. Robert Belle Burke. Philadelphia: University of Pennsylvania Press.

[CR5] Bashford, Alison. 2010. Epilogue: Where Did Eugenics Go? In *The Oxford Handbook of the History of Eugenics*, eds. Alison Bashford and Philippa Levine, 539–558. Oxford: Oxford University Press.

[CR6] Bostrom, Nick. 2024. *Deep Utopia: Life and Meaning in a Solved World*. Washington, D.C.: Ideapress Publishing.

[CR7] Brodman, James. 2009. *Charity and Religion in Medieval Europe*. Washington, D.C.: Catholic University of America.

[CR8] Cady, Linell E. Religion the Technowonderland of Transhumanism. In *Building Better Humans? Refocusing the Debate on Transhumanism*, Hava Tirosh-Samuelson Kenneth L., and Mossman. 2011. 83–104. Frankfurt am Main: Peter Lang.

[CR9] Carr, Nicholas G. 2011. *The Shallows: How the Internet is Changing the Way We Think, Read and Remember*. London: Atlantic.

[CR10] Chung, Yeuhsten Juliette. 2014. Better Science and Better Race? Social Darwinism and Chinese Eugenics. *Isis* 1054:793–802. 10.1086/679426 25665386

[CR11] Courtenay, William J. 2001. Curers of Body and Soul: Medical Doctors as Theologians. In *Religion and Medicine in the Middle Ages*, eds. Peter Biller and Joseph Ziegler, 69–75. Woodbridge: York Medieval Press.

[CR12] Crisciani, Chiara. 2009. Premesse e promesse di lunga vita: tra teologia e pratica terapeutica (secoli XIII-XIV). In *Vita longa. Vecchiaia e durata della vita, nella tradizione medica e aristotelica antica e medievale*, ed. C. Crisciani, L. Repici, and P. B. Rossi. 61–86. Florence: SISMEL. Micrologus 33.

[CR13] Crisciani, Chiara. 2010. Introduction. In *Arnaldo de Villanova, De humido radicale*, ed. Michael McVaugh. 319–571. Barcelona: Publicacions de la Universitat de Barcelona.

[CR14] Crisciani, Chiara. 2013. Prolungamento della vita: medicina e teologia (secoli xiii e xiv). In *Médecine et religion: Compétitions, collaborations, conflits (XIIe-XXe siècles)*, ed. Maria P. Donato. 87–113. Rome: École française de Rome. Collection de l’École française de Rome 476.

[CR15] Daly, Todd. 2021. *Chasing Methuselah: Theology, the Body, and Slowing Human Aging*. Eugene, Or.: Cascade Books.

[CR57] de Gray, Aubrey. 2007. Old People Are People Too: Why it is Our Duty to Fight Aging to the Death. Cato Unbound: A Journal of Debate. https://www.cato-unbound.org/2007/12/03/aubrey-de-grey/old-people-are-people-too-why-it-our-duty-fight-aging-death/

[CR16] De Rijk, L. M. 1967. Some Notes on the Twelfth Century Topic of the Three (Four) Human Evils and of Science, Virtue and Techniques as their Remedies. *Vivarium* 5:8–15. 10.1163/156853467x00023

[CR17] French, Roger K., and Andrew Cunningham. 1996. *Before Science: The Invention of the Friars’ Natural Philosophy*. Aldershot: Scholar Press.

[CR18] Friedmann, Theodore. 2019. Genetic Therapies, Human Genetic Enhancement, and… Eugenics? *Gene Therapy* 269:351–353. 10.1038/s41434-019-0088-1 31273325

[CR19] Getz, Faye. 1997. Roger Bacon and Medicine: The Paradox of the Forbidden Fruit and the Secrets of Long Life. In *Roger Bacon and the Sciences: Commemorative Essays*, ed. Jeremiah Hackett. 337–64: Leiden: Brill.

[CR20] Giubilini, Alberto, and Sagar Sanyal. 2015. The Ethics of Human Enhancement. *Philosophy Compass* 10(4):233–243. 10.1111/phc3.12208

[CR21] Grant, Edward. 1996. *The foundations of modern science in the middle ages: Their religious, institutional, and intellectual contexts*. Cambridge: Cambridge University Press.

[CR22] Grim, Stephen R. 2017. Why Study History? On its Epistemic Benefits and its Relation to the Sciences. *Philosophy* 92(3):399–420.

[CR23] Huxley, Julian. 1941a. Eugenics and Society. In *Man Stands Alone*, ed. Julian Huxley, 34–84.. New York: Harper and Row.

[CR24] Huxley, Julian. 1941b. Life Can Be Worth Living. In *Man Stands Alone*, ed. Julian Huxley. 291–300. New York: Harper and Row.

[CR25] Huxley, Julian. 1941c. Scientific Humanism. In *Man Stands Alone*, ed. Julian Huxley. 260–276. New York: Harper and Row.

[CR26] Jacquart, Danielle. 2010. Medicine and Theology. In *Crossing Boundaries at Medieval Universities*, ed. Spencer E. Young. 213–226. Leiden: Brill.

[CR27] Jordan, Mark D. 1988. Medicine and Natural Philosophy in Aquinas. In *Thomas von Aquin: Werk und Wirkung im Licht Neuerer Forschungen*, ed. Albert Zimmermann, 233–46. Berlin: Walter de Gruyter.

[CR28] Kass, Leon. 2002*. Life, Liberty, and Defense of Dignity: Challenges in Bioethics*. San Francisco: Encounter Books.

[CR29] Kosinski, Michal. 2021. Facial Recognition Technology Can Expose Political Orientation from Naturalistic Facial Images. *Nature: Scientific Reports* 11:100. 10.1038/s41598-020-79310-1 PMC7801376 33431957

[CR30] Lerner, Barron, and Arthur Caplan. Judging the Past: How History Should Inform Bioethics. *Annals of Internal Medicine*(164)8: 553–557. 10.7326/M15-2642 27089070

[CR31] Linden, Ingemar Patrick. 2022. *The Case Against Death*. Cambridge, Ma.: MIT Press.

[CR32] Yuancheng, Ryan Lu, Tian, Xiao, and David Sinclair. 2023. The Information Theory of Aging. *Nature Aging* 312:1486–1499. 10.1038/s43587-023-00527-6 38102202

[CR33] Lyreskog, David, Zohny, Hazem, Singh, Ilina, and Julian Savulescu. 2023. The Ethics of Thinking with Machines: Brain-Computer Interfaces in the Era of Artificial Intelligence. *International Journal of Chinese & Comparative Philosophy of Medicine* 212:11–34. 10.24112/ijccpm.212676

[CR34] McVaugh, Michael. 1974. The *humidum radicale* in Thirteenth-Century Medicine. *Traditio* 30:259–283. 10.1017/s0362152900006528 11633405

[CR35] Meilander, Gilbert. 2013. *Should We Live Forever? The Ethical Ambiguities of Aging*. Grand Rapids: William B. Eerdmans Publishing Company.

[CR36] Noble, David F. 1997. *The Religion of Technology: The Divinity of Man and the Spirit of Invention*. Toronto: University of Toronto.

[CR37] Paravicini Bagliani, Agostino. 2000. *The Pope’s Body*. Chicago: University of Chicago Press.

[CR38] Paravicini Bagliani, Agostino. 2003. Ruggero Bacone e l’alchimia di lunga vita. Riflessioni sui testi. In *Alchimia e medicina nel Medioevo*, eds. Chiara Crisciani and Agostino Paravicini Bagliani, 33–54. Florence: SISMEL.

[CR39] Paul, Diane. 2014. What Was Wrong with Eugenics? Conflicting Narratives and Disputed Interpretations. *Science and Education* 232:259–271. 10.1007/s11191-012-9556-3

[CR40] Pernick, Martin. 2008. Bioethics and History. In *The Cambridge World History of Medical Ethics*, eds. Robert Baker and Laurence McCullough, 16–20. Cambridge: Cambridge University Press.

[CR41] President’s Council on Bioethics. 2003. *Beyond Therapy: Biotechnology and the Pursuit of Happiness*. LSU Medical and Public Health Site. https://biotech.law.lsu.edu/research/pbc/reports/beyondtherapy/

[CR42] Ricoeur, Paul. 1990. *Time and Narrative**Vol. 1*.Chicago: University of Chicago Press.

[CR43] Sandel, Michael. 2007. *The Case against Perfection: Ethics in the Age of Genetic Engineering*. Cambridge, Mass.: Belknap Press of Harvard University.

[CR44] Sohn, Sei Yon, Rees, Philippa, Wildridge, Bethany, Kalk, Nicola, and Ben Carter. 2019. Prevalence of Problematic Smartphone Usage and Associated Mental Health Outcomes Amongst Children and Young People: A Systematic Review. BMC Psychiatry (19)356.:1-10. 10.1186/s12888-019-2350-x PMC6883663 31779637

[CR45] Southern, Richard W. 1970. Medieval Humanism. In R. W. Southern, *Medieval Humanism and Other Studies*, 29–60. Oxford: Basil Blackwell.

[CR46] Sparrow, Robert. 2011. A Not-So-New Eugenics. *The Hastings Center Report* 41(1):32–42 10.1002/j.1552-146x.2011.tb00098.x 21329104

[CR47] Turda, Marius, and Aaron Gillette. 2014. *Latin Eugenics in Comparative Perspective*. London: Bloomsbury Academic.

[CR48] Wang, Yilun, and Michal Kosinski. 2018. Deep Neural Networks are more Accurate than Humans at Detecting Sexual Orientation from Facial Images. *Journal of Personality and Social Psychology* 114(2):246–257. 10.1037/pspa0000098 29389215

[CR49] Wilson, Duncan. 2011. What Can History Do for Bioethics? *Bioethics* (27)4: 215–233. 10.1111/j.1467-8519.2011.01933.x PMC3654172 22150828

[CR50] Wisniewski, Robert. 2020. *Christian Divination in Late Antiquity*. Amsterdam: Amsterdam University.

[CR51] Wright, N. T. 2019. *History and Natural Theology: Jesus and the Promise of Natural Theology*. London: SPCK.

[CR52] Young, George. 2012. *The Russian Cosmists: The Esoteric Futurism of Nikolai Fedorov and his Followers*. New York: Oxford University Press.

[CR53] Ziegler, Joseph. 1999. Ut dicunt medici: Medical Knowledge and Theological Debates in the Second Half of the Thirteenth Century. *Bulletin of the History of Medicine* 73:208–237. 10.1353/bhm.1999.0091 10379088

[CR54] Ziegler, Joseph. 2001. Medicine and Immortality in Terrestrial Paradise. In *Religion and Medicine in the Middle Ages*, eds. Peter Biller and Joseph Ziegler, 201–42. Woodbridge: York Medieval Press.

[CR55] Ziegler, Joseph. 2017. Engelbert of Admont and the Longevity of the Antediluvians, c. 1300. In *Summa doctrina et certa experientia: studi su medicina e filosofia per Chiara Crisciani*, ed. Gabriella Zuccolin. 313–36. Florence: SISMEL. Micrologus library 79.

[CR56] Ziegler, Joseph. 2023. Complexio and the Transformation of Learned Physiognomy ca. 1200-ca. 1500. *Early Science and Medicine* 28(3–5): 472–497. 10.1163/15733823-20230083

